# Both the serum AFP test and AFP/GPC3/SALL4 immunohistochemistry are beneficial for predicting the prognosis of gastric adenocarcinoma

**DOI:** 10.1186/s12876-021-01986-0

**Published:** 2021-10-27

**Authors:** Bingzhi Wang, Yibin Xie, Li Zheng, Xiaohao Zheng, Jia Gao, Xiuyun Liu, Yanling Yuan, Zhuo Li, Ning Lu, Liyan Xue

**Affiliations:** 1 Department of Pathology, National Cancer Center/National Clinical Research Center for Cancer/Cancer Hospital, Chinese Academy of Medical Sciences and Peking Union Medical College, Beijing, 100021 China; 2 Department of Abdominal Surgical Oncology, National Cancer Center/National Clinical Research Center for Cancer/Cancer Hospital, Chinese Academy of Medical Sciences and Peking Union Medical College, Beijing, 100021 China; 3 Department of General Surgery, the First People’s Hospital of Dongcheng District, Beijing, 100075 China; 4 Department of Clinical Laboratory, National Cancer Center/National Clinical Research Center for Cancer/Cancer Hospital, Chinese Academy of Medical Sciences and Peking Union Medical College, Beijing, 100021 China

**Keywords:** Gastric cancer, Pathology, Biomarker, Metastases

## Abstract

**Background:**

Both gastric adenocarcinoma with primitive enterocyte phenotype (GAPEP) (including hepatoid adenocarcinoma) and alpha-fetoprotein (AFP)-producing gastric adenocarcinoma have poor prognoses. However, the value of the serum AFP test and AFP/glypican-3 (GPC3)/spalt-like transcription factor 4 (SALL4) immunohistochemistry is still not clear, and these two methods have not yet been thoroughly compared.

**Methods:**

We collected 421 consecutive non-neoadjuvant surgically or endoscopically resected gastric adenocarcinoma patients with serum AFP results before surgery (group A). We divided these cases into serum AFP-high (sAFP-H) and serum AFP-normal (sAFP-N) by serum AFP levels, and into GAPEP (expressing AFP, GPC3, or SALL4) and non-GAPEP (nGAPEP) by AFP/GPC3/SALL4 immunohistochemistry results. We also collected 12 non-resected gastric adenocarcinoma patients with serum AFP ≥ 7 ng/mL before treatment (group B). We analyzed these patients’ clinicopathological characteristics and prognoses.

**Results:**

Seventeen (4.04%) patients in group A were sAFP-H. These patients were younger and mainly had tubular adenocarcinoma with later pT (*P* = 0.014) and pN (*P* = 0.047) categories and more lymphovascular invasion (*P* < 0.001), perineural spread (*P* = 0.008), and metastases or recurrence (*P* < 0.001). For immunohistochemistry, 34 (8.08%) cases were GAPEP, and GAPEP cases also had later pT categories than nGAPEP cases (*P* = 0.001). Most group B patients with elevated serum AFP (especially > 1000 ng/mL) had simultaneous metastases, mainly liver metastases. Both the serological method and immunohistochemical method were useful for predicting prognosis (AUC _sAFP_ = 0.625, AUC _A/G/S-IHC_ = 0.723, z statistic = 1.726, *P* = 0.084). The serum AFP level (especially > 1000 ng/mL) is more specific (100%), and immunohistochemistry is more sensitive (50%).

**Conclusion:**

Both the serum AFP level and immunohistochemical expression of AFP/GPC3/SALL4 can be used to indicate a poor prognosis for gastric adenocarcinoma.

## Background

Gastric cancer is one of the most common malignant tumors and a heterogeneous malignant tumor [1]. Although most gastric cancers are adenocarcinomas, their biological behavior and prognosis are significantly different. The detection of serum biomarkers is helpful for predicting the prognosis of gastric adenocarcinomas [2, 3]. One of the most widely studied markers is serum alpha-fetoprotein (sAFP). In 1970, Bourreille et al. proposed the concept of hepatoid adenocarcinoma (HAC) based on morphology and found that this subtype was often accompanied by elevated sAFP and more likely to have liver metastases [4, 5]. Subsequent studies also found that gastric adenocarcinoma with enteroblastic differentiation (GAED) and yolk-sac tumor-like carcinoma had similar characteristics [6, 7]. Successive studies concluded that regardless of whether there was a special pathological morphology, cases of positive AFP immunohistochemistry or elevated sAFP had a suggestive risk of progression, collectively referred to as "AFP-producing gastric adenocarcinoma" [8, 9]. This concept suggests transformation from morphology to molecular biology. With the application of more immunohistochemical markers, Yamazawa et al. found that AFP, glypican-3 (GPC3) and spalt-like transcription factor 4 (SALL4) immunohistochemistry (A/G/S-IHC) outcomes had similar effects. Patients expressing at least one of these proteins (AFP, GPC3 or SALL4) had a poor prognosis and frequently exhibited liver metastases regardless of morphology. They called this subtype “gastric adenocarcinoma with primitive enterocyte phenotype (GAPEP)” [10].

However, several unresolved problems remain in previous studies. The relationship between GAPEP and sAFP level is not clear, and no studies have compared the sAFP level and A/G/S-IHC results in gastric adenocarcinoma. The cutoff value for sAFP elevation is not uniform, and the specific significance of the degree of sAFP elevation is not clear [11]. In most hospitals, the sAFP test and A/G/S-IHC test are not routine tests for gastric carcinoma patients, though both methods are easy to perform widely.

In this study, we collected gastric adenocarcinoma cases with definite sAFP levels and then analyzed their clinicopathological characteristics and immunohistochemical results of AFP, GPC3, and SALL4. We tried to determine the value of sAFP and A/G/S-IHC in the diagnosis and treatment of gastric adenocarcinoma.

## Materials and methods

### Enrollment criteria

For enrollment, patients must have met the following conditions: admission to the National Cancer Center/National Clinical Research Center for Cancer/Cancer Hospital, Chinese Academy of Medical Sciences and Peking Union Medical College between December 2016 and December 2018 and without history of germ cell tumors, primary hepatocellular carcinoma or active hepatitis. We also excluded the cases with probable liver cirrhosis according to imaging and blood biochemistry reports. Two groups were established. Group A included 421 cases consecutive non-neoadjuvant surgically (392 cases) or endoscopically (29 cases) resected gastric adenocarcinoma patients with sAFP results before resection. Group B included 12 non-resected gastric adenocarcinoma patients with serum AFP ≥ 7 ng/mL before treatment (group B).

### Clinicopathological characteristics and follow-up

The clinicopathological information collected from all patients included sex, age, concurrent metastases, primary sAFP levels, Lauren classification and detailed histological classification (based on the Japanese Gastric Cancer Association classification [12]: tub1 = well differentiated tubular type, tub2 = moderately differentiated tubular type, por1 = solid type, por2 = poorly cohesive type, sig = signet-ring cell type, muc = mucin type, and other types). For group A, we also collected macroscopic classification, tumor location, tumor and node categories (American Joint Committee on Cancer (AJCC) 8th edition [13]), lymphovascular invasion (LVI), and perineural spread (PNS). Follow-up information included the location of postoperative recurrence in group A cases and the location of tumor progression in group B cases. The day of last follow-up was October 1, 2020.

### Serum AFP test

Serum AFP was measured by an electrochemiluminescence assay using a Cobas e602 immunoassay analyzer (Roche Diagnostics, Germany) and the Elecsys AFP Kit (Roche Diagnostics, Germany). According to sAFP level before resection, cases in group A were divided into two groups: serum AFP-high (sAFP-H, sAFP ≥ 7 ng/mL) and serum AFP-normal (sAFP-N, sAFP < 7 ng/mL). The sAFP-H variation before and after resection was also recorded.

### Immunohistochemical test

Immunohistochemical staining was performed with primary antibodies against AFP (1:100, Clone 1E4; Gene Tech Company Limited, Shanghai, China), GPC3 (1:100, Clone 1G12, ZSGB-Bio Company Limited, Beijing, China), and SALL4 (1:100, Clone 6E3, ZSGB-Bio Company Limited, Beijing, China). All immunohistochemical staining was performed in Ventana Benchmark XT. The results were considered positive when ≥ 1% cytoplasmic staining for AFP and GPC3 and ≥ 10% nuclear staining for SALL4 [14–16]. All standard and measured histopathological variables and all immunohistochemistry slides were first reviewed and graded independently by two authors (BW and LX), and then discordant cases were reviewed jointly until a consensus was reached. We divided the group A cases by the AFP/GPC3/SALL4 results into gastric cancer with primitive enterocyte phenotype (GAPEP, at least one of three was positive) and non-GAPEP (nGAPEP, all three markers were negative).

### Statistical analysis

The clinicopathological characteristics and prognoses in groups A and B were analyzed. We performed 1:1 propensity score matching (PSM) in group A (both sAFP-N vs*.* sAFP-H and GAPEP vs*.* nGAPEP) to compare the Progression Free Survival (PFS), and the PSM predictors included age, Lauren classification, and T and N categories (match tolerance < 0.2, random seed to verify the reliability). Metastases in group B, including simultaneous metastases and metachronous metastases, were analyzed. The heatmap of A/G/S-IHC of sAFP-H cases was drawn by Prism 8 software (GraphPad Software Ltd., San Diego, USA). The relationship between immunohistochemistry and a high risk of disease progression (simultaneous metastases or postoperative metastases within three years) was analyzed. The comparison of count data was made by the chi-square test with a significance level of 0.05 on two-tailed P-values. SPSS (Statistical Product and Service Solutions, IBM Corp., NY, United States) 25.0 software was used for statistical analysis. Receiver operating characteristic (ROC) curves were plotted, and the area under the curve (AUC) and z-test were calculated.

## Results

### Clinicopathological characteristics in group A

**Fig. 1:**
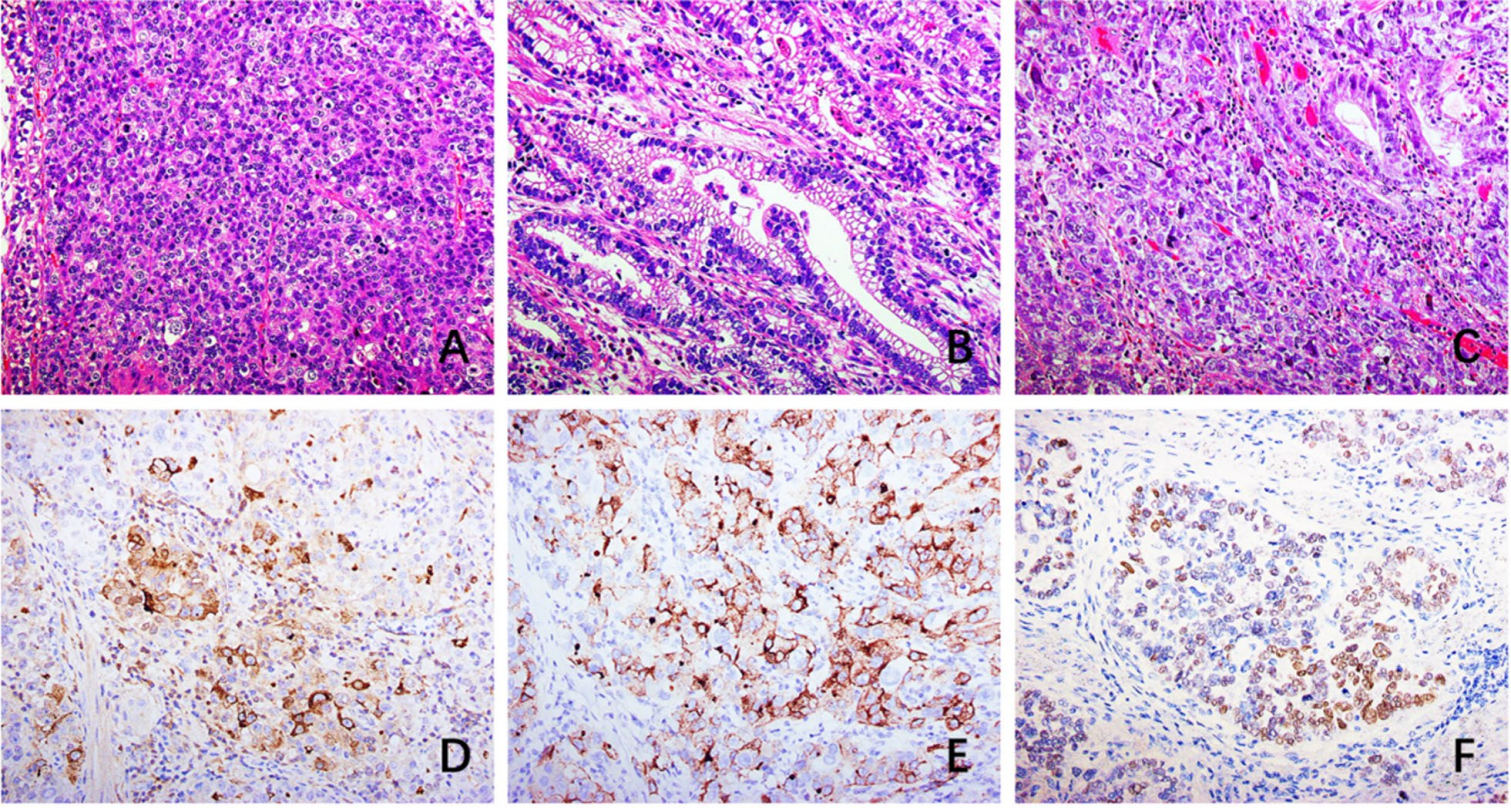
**A** Hepatoid carcinoma area. **B** Adenocarcinoma with enteroblastic differentiation. **C** Moderately poorly differentiated adenocarcinomas with positive AFP (**D**), GPC3 (**E**) and SALL4 (**F**). All X200

**Fig. 2:**
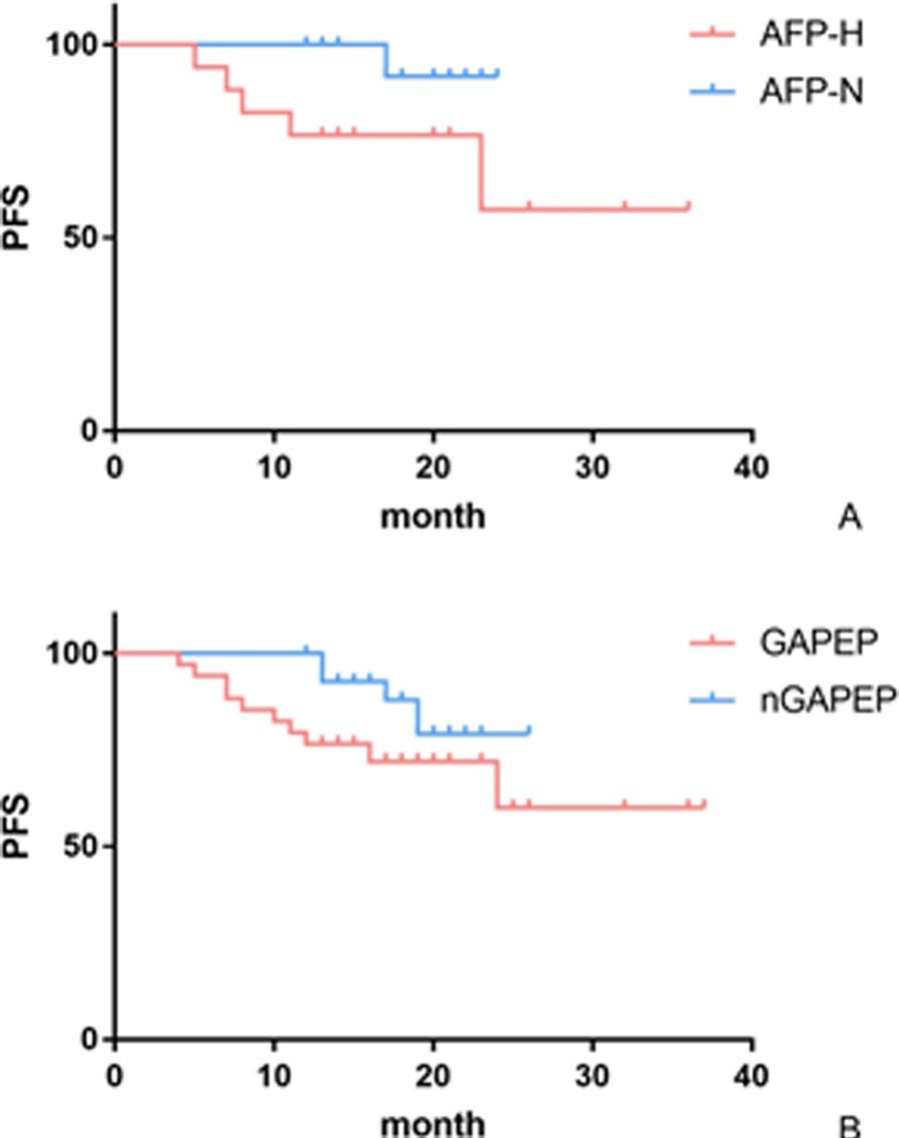
Progression-free survival (PFS) curves (months) after 1:1 propensity score matching (matching predictors included the ages, Lauren classification, T and N categories). **A** sAFP-N versus sAFP-H (*P* = 0.048). **B** GAPEP versus nGAPEP (*P* = 0.035)

According to sAFP level, 404 (96.19%) cases were assigned to the sAFP-N group, and 17 (3.81%) cases were assigned to the sAFP-H group (Table 1). When the cases were divided by a cutoff age of 50 years, significantly more patients aged < 50 years were noted in the sAFP-H group than those in the sAFP-N group (*P* = 0.023). Definite differences in both Lauren classification (*P* = 0.030) and JCGA histological classification (*P* = 0.021) were identified. Regarding the JGCA classification, the sAFP-H cases mainly composed of tub2 type (8, 47.06%) and por1 type (4, 23.53%). More por2 type were observed in the sAFP-N cases. Notably, few cases of special subtypes were noted in the sAFP-H group: only 2 cases were HAC, 1 case was GAED, and the other cases were mostly moderately or poorly differentiated tubular adenocarcinoma with no special morphological features (Fig. 1). There were significant differences in T categories (*P* = 0.014). More T4 cases were in the sAFP-H cases (10, 58.82%) than those in the sAFP-N cases (150, 37.13%). Significant difference in N categories was identified (*P* = 0.047). The sAFP-H group had significantly more N3 cases than the sAFP-N group (52.94% vs 29.60%). LVI (76.47% vs 35.15%, *P* < 0.001) and PNS (64.71% vs 33.42%, *P* = 0.008) were significantly higher in the sAFP-H group than that in the sAFP-N group, respectively. The sAFP-H group had significantly more postoperative recurrence or metastases than the sAFP-N group (23.53% vs 5.66%, *P* < 0.001). Among the sAFP-H group, 3 patients had liver metastasis, and 1 patient had recurrence of the remnant stomach at one year after surgery. After 1:1 PSM, sAFP-H patients also had worse PFS than sAFP-N patients (*P* = 0.048) (Fig. 2A). There were no significant differences in sex (*P* = 0.779), location (*P* = 0.164), or macroscopic classification (*P* = 0.064).

**Table 1:** Comparison of clinicopathological characteristics between sAFP-N *vs*. sAFP-H and between nGAPEP *vs.* GAPEP in the surgical cases (group A)

		sAFP-N	sAFP-H	*P*	nGAPEP	GAPEP	*P*
Age	≤ 50	93 (23.02%)	8 (47.06%)	0.023	91 (23.51%)	10 (29.41%)	0.440
	> 50	311 (76.98%)	9 (52.94%)		296 (76.49%)	24 (70.59%)	
Sex	Male	272 (67.33%)	12 (70.59%)	0.779	259 (66.93%)	25 (73.53%)	0.431
	Female	132 (32.67%)	5 (29.31%)		128 (33.07%)	9 (26.47%)	
Location^*^	Proximal	64 (16.04%)	2 (11.76%)	0.164	60 (15.67%)	6 (18.18%)	0.359
	Distal	275 (68.92%)	15 (88.24%)		265 (69.19%)	25 (75.76%)	
	Overlapping	60 (15.04%)	0 (0.00%)		58 (15.14%)	2 (6.06%)	
Lauren classification	Intestinal	88 (21.78%)	8 (47.06%)	0.030	83 (21.45%)	13 (38.24%)	0.081
	Diffuse	262 (64.85%)	6 (35.29%)		251 (64.86%)	17 (50.00%)	
	Mixed	54 (13.37%)	3 (17.65%)		53 (13.69%)	4 (11.76%)	
Histological classification^**^	Tub1	10 (2.48%)	0 (0.00%)	0.021	10 (2.58%)	0 (0%)	0.009
	Tub2	72 (17.82%)	8 (47.06%)		68 (17.57%)	12 (35.29%)	
	Muc	6 (1.49%)	0 (0.00%)		5 (1.29%)	1 (2.94%)	
	Sig	139 (34.41%)	0 (0.00%)		137 (35.40%)	2 (5.88%)	
	Por1	62 (15.35%)	4 (23.53%)		58 (14.99%)	8 (23.53%)	
	Por2	61 (15.10%)	2 (11.76%)		56 (14.47%)	7 (20.59%)	
	Others	54 (13.37%)	3 (17.65%)		53 (13.70%)	4 (11.76%)	
T category	T1	142 (35.15%)	0 (0.00%)	0.014	140 (36.18%)	2 (5.88%)	0.001
	T2	57 (14.11%)	5 (29.41%)		52 (13.44%)	10 (29.41%)	
	T3	55 (13.61%)	2 (11.76%)		54 (13.95%)	3 (8.82%)	
	T4	150 (37.13%)	10 (58.82%)		141 (36.43%)	19 (55.88%)	
N category	N0	150 (40.00%)	3 (17.65%)	0.047	141 (39.39%)	12 (35.29%)	0.152
	N1	63 (16.80%)	1 (5.88%)		62 (17.32%)	2 (5.88%)	
	N2	51 (13.60%)	4 (23.53%)		47 (13.13%)	8 (23.53%)	
	N3	111 (29.60%)	9 (52.94%)		108 (30.17%)	12 (35.29%)	
LVI	No	262 (64.85%)	4 (23.53%)	0.001	249 (64.34%)	17 (50.00%)	0.096
	Yes	142 (35.15%)	13 (76.47%)		138 (35.66%)	17 (50.00%)	
PNS	No	269 (66.58%)	6 (35.29%)	0.008	257 (66.41%)	18 (52.94%)	0.114
	Yes	135 (33.42%)	11 (64.71%)		130 (33.59%)	16 (47.06%)	
Postoperative recurrence/metastasis	No	200 (94.34%)	13 (76.47%)	< 0.001	379 (97.93%)	26 (76.47%)	< 0.001
	Yes	12 (5.66%)	4 (23.53%)		8 (2.07%)	8 (23.53%)	

LVI, lymphovascular invasion; PNS, perineural spread

*Proximal = tumor in upper 1/2 with a proximal gastrectomy; dismal = tumor in lower 1/2 with a dismal gastrectomy; overlapping = large tumor or linitis plastica with a total gastrectomy

**Based on the Japanese Gastric Cancer Association classification, tub1 = well differentiated tubular type, tub2 = moderately differentiated tubular type, por1 = solid type, por2 = poorly cohesive type, sig = signet-ring cell type, muc = mucin type, and other types

By the A/G/S-IHC results, 34/421 (8.08%) cases were GAPEP. The positive rate of AFP was 4.28% (18/421), the positive rate of GPC3 was 4.51% (19/421), and the positive rate of SALL4 was 2.85% (12/421). The GAPEP cases also showed similar characteristics as sAFP-H cases, especially in JGCA classification (*P* = 0.009) and T category (*P* = 0.001). The GAPEP group had significantly more postoperative recurrence or metastases than the nGAPEP group (23.53% vs 2.07%, *P* < 0.001) 1 year after surgery. After 1:1 PSM, GAPEP patients also had worse survival than nGAPEP patients (*P* = 0.035) (Fig. 2B).

**Fig. 3:**
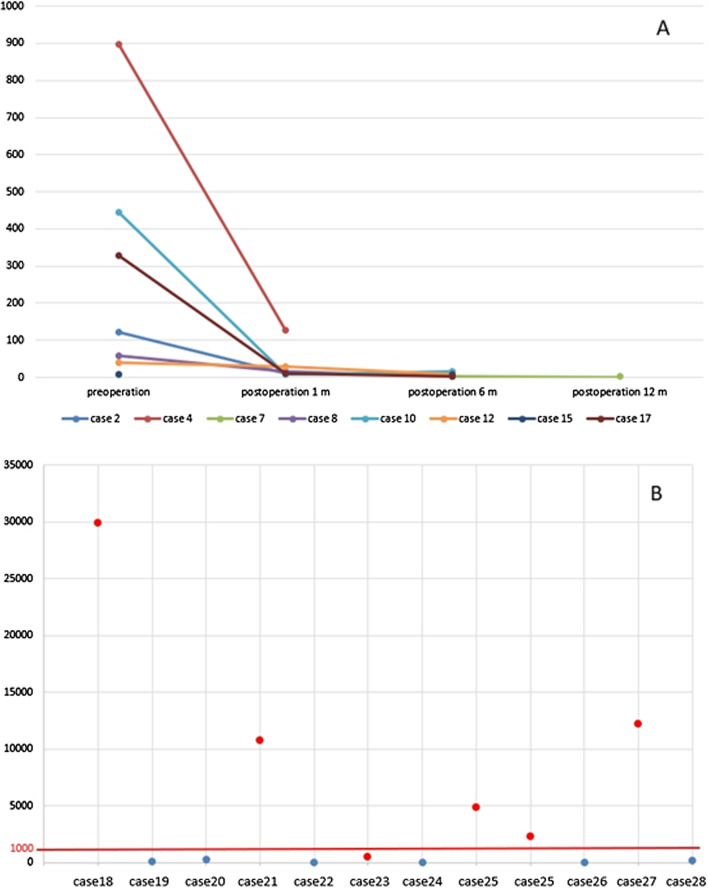
**A** The serum AFP value (ng/mL) varied from the preoperative period to the postoperative period in 8 cases in group A. **B** The serum AFP value (ng/mL) of 12 cases in group B. Each dot represents a case, and the red dot indicates liver metastases

Serum AFP was re-examined in 7 sAFP-H patients after surgery. The sAFP levels in these 7 patients were significantly decreased after surgery, and most fell in the normal range within one year after surgery (Fig. 3A).

### Clinicopathological characteristics in group B

In group B, a total of 7 cases had simultaneous metastases (58.33%), of which 4 cases were liver metastases, 2 cases were ovarian metastases, and 1 case was liver metastases accompanied by lung metastases at the same time. The rate of liver metastases reached 41.67%. Two patients had metachronous liver metastases during follow-up. The total liver metastasis rate reached 58.33%. For the A/G/S-IHC results, 11/12 cases were GAPEP.

The clinicopathological characteristics of the 29 sAFP-H cases in groups A and B are shown in Table 2. Interestingly, all patients with sAFP > 1000 ng/mL in groups A and B had liver metastases, most of whom had liver metastases at diagnosis, while only one case had metachronous metastasis (Fig. 3B).

**Table 2:** Clinicopathological characteristics of the sAFP-H cases in groups A and B

Case	Patient group	Age	Sex	Location	Macroscopic	Diameter (cm)	Premier sAFP (ng/mL)	Simultaneous metastasis	Metachronous metastasis/recurrence	JGCA type	pT	pN	LVI	PNS	AFP	GPC3	SALL4
1	A	69	M	Distal	3	4	629			por1	T2	N0	−	−	+	+	+
2	A	44	M	Distal	3	6	498.3			por1	T3	N2	+	+	+	+	−
3	A	57	M	Distal	2	5.5	15.36			tub2	T4	N3	+	+	+	−	+
4	A	65	F	Distal	2	4	25.99			others	T2	N3	+	−	+	−	−
5	A	43	M	Distal	2	6	14.45			por2	T4	N3	+	+	+	+	−
6	A	56	M	Distal	2	7.3	55.03		Liver	por1*	T4	N3	+	−	+	+	+
7	A	59	F	Distal	3	3.5	897.3		Liver	tub2	T4	N3	+	+	+	+	+
8	A	63	M	Distal	2	3	7.74			tub2	T2	N0	+	−	+	+	+
9	A	40	F	Distal	3	6	169.8			tub2	T4	N2	+	+	+	−	−
10	A	48	M	Distal	2	5	58.15		Liver	tub2**	T4	N3	+	+	+	−	−
11	A	66	M	Distal	2	3	101			others	T2	N2	−	+	+	−	−
12	A	41	M	Proximal	2	6.2	443.6			por1*	T4	N2	+	+	+	−	+
13	A	45	F	Distal	2	4	72.58			tub2	T2	N3	−	−	+	−	−
14	A	64	M	Distal	2	4	9.18			tub2	T4	N3	+	−	+	−	−
15	A	47	F	Distal	2	4.5	9.33		Stump recurrence	por2	T4	N0	−	+	+	−	−
16	A	60	M	Distal	2	5	204.9			tub2	T3	N1	+	+	+	+	−
17	A	50	M	Proximal	1	9	329.5			others	T4	N3	+	+	+	−	+
18	B	62	M	Proximal			29,854	Liver		por1					−	−	+
19	B	61	M	Proximal			121.4			tub2					+	+	+
20	B	64	M	Proximal			236.8	Ovary		tub2					−	−	+
21	B	65	M	Distal			10,777	Liver		por1					+	+	+
22	B	49	M	Proximal			31.54	Ovary		tub2					+	−	+
23	B	66	F	Distal			444.8		Liver	por1					+	+	+
24	B	62	F	Proximal			25.24			por1					−	−	−
25	B	56	M	Distal			4839	Liver		tub2					−	+	+
26	B	66	M	Distal			2268		Liver	tub2					+	+	+
27	B	40	F	Proximal			40.34	Liver		tub2					−	−	+
28	B	59	M	Distal			12,226	Liver		tub2					+	−	+
29	B	53	M	Proximal			144.8			por1					+	+	+

*With hepatoid carcinoma component

**With enteroblastic component

### Comparison and correlation of immunohistochemistry and sAFP test

Serum AFP-H patients had significantly higher positive rates of AFP, GPC3, and SALL4 than sAFP-N patients (Table 3). Serum AFP-H was significantly related to GAPEP (*P* < 0.001). Notably, more high-risk cases were detected by immunohistochemistry, 17 surgical cases of GAPEP with normal sAFP were identified, 4 of which had postoperative recurrence or metastasis (2 cases of recurrence in gastric remnants, 1 case of liver metastasis, and 1 case of lung metastasis).

**Table 3:** Correlation between serum AFP value and immunohistochemical results

		sAFP-N	sAFP-H	*P*
AFP	(−)	403 (99.75%)	0	< 0.001
	(+)	1 (0.25%)	17 (100.00%)	
GPC3	(−)	392 (97.03%)	10 (58.82%)	< 0.001
	(+)	12 (2.97%)	7 (41.18%)	
SALL4	(−)	399 (98.76%)	10 (58.82%)	< 0.001
	(+)	5 (1.24%)	7 (41.18%)	
GAPEP	(−)	387 (95.79%)	0	< 0.001
	(+)	17 (4.21%)	17 (100%)	

**Fig. 4:**
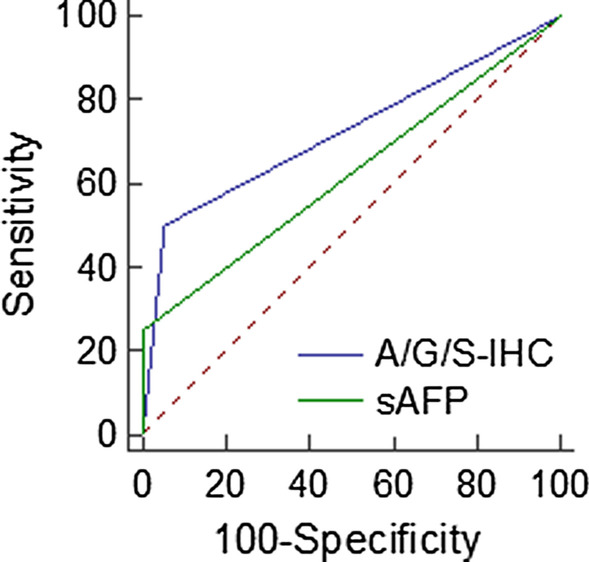
ROC curves of the two methods individually to predict progression free survival in group A (AUC _sAFP_ = 0.625, AUC _A/G/S-IHC_ = 0.723, z statistic = 1.726, *P* = 0.084)

**Fig. 5:**
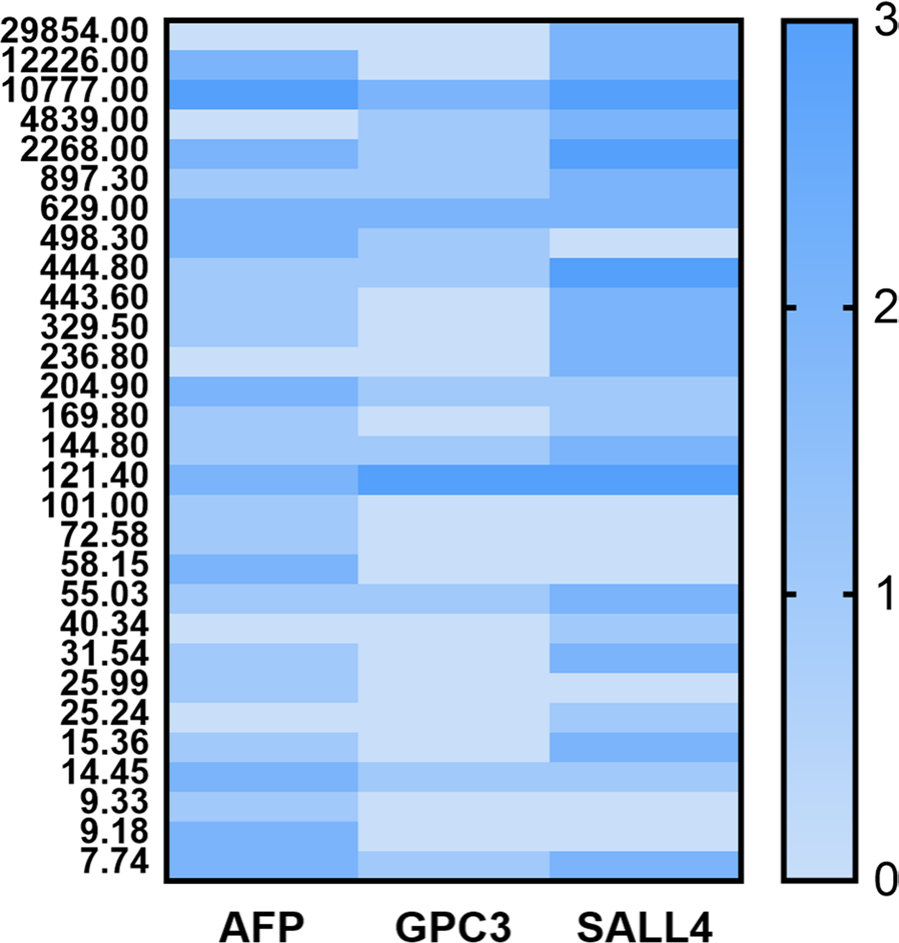
Heatmap of all sAFP-H cases in groups A and B. Horizontal axis, AFP, GPC3 and SALL4 results. Vertical axis, sAFP-H cases in descending order of sAFP level

With a high risk of disease progression as the observation index, both methods had good efficacy (AUC _sAFP_ = 0.625, AUC _A/G/S-IHC_ = 0.723, z statistic = 1.726, *P* = 0.084) (Fig. 4). The serum test had higher specificity (100% vs 94.5%) while the immunohistochemical test had higher sensitivity (50% vs 25%). We also drew a heatmap of all sAFP-H cases (Fig. 5).

## Discussion

In this study, we compared the serum AFP test and immunohistochemical test of GAPEP to find the value of the two methods in clinical diagnosis. The proportion of sAFP-H patients in group A was 1.82%, which was slightly lower than the number reported in the literature [17]. The reason might be this study is a retrospective research and not every patient underwent the sAFP test in our hospital.

Though some studies argued the predictive value of serum AFP [18], most previous studies have confirmed that the prognosis of even early gastric adenocarcinoma with elevated serum AFP is poor [19]. It is believed that the production of sAFP is more likely to be caused by gastric cancer itself. AFP produced by the tumor itself that leads to an increase in the serum, and sAFP loses its source after tumor resection and then rapidly decreases, similar to our result (Fig. 3A). Some cases (3/8) with sAFP dropping after surgery had progression later, which might reflect the aggressive characteristics. However, the quantity of cases with both pre- and post-operation sAFP was limited. Another phenomenon that must be noted is that patients with extremely high sAFP values (> 1000 ng/mL) had liver metastases at diagnosis, which may reflect high tumor burden. It is suggested that the sAFP test could be an important signal of liver metastasis. This study did not include biopsy cases with normal sAFP as a control for group B. Serum AFP-H patients often have simultaneous metastases at diagnosis, which is sufficient to demonstrate the biological characteristics. Further controlled studies of sAFP-H and sAFP-N in biopsy cases are necessary to better understand the implication of sAFP-H.

The mechanism of the special biological behavior of liver metastasis has been explored in some studies [20, 21]. One reason may be that this tumor produces many tumor thrombi, and the liver and lungs are vital for blood circulation, so the tumor thrombi easily spread to the liver through the blood [22]. Another possible reason is that the tumor itself activates certain molecular pathways, so it easily colonizes and grows in the liver [23, 24]. From the perspective of embryonic development, the liver, gastrointestinal tract, and lung originate from the endoderm, and those organs develop closely in a relatively long period [25, 26]. The lineage of primitive epithelial differentiated tumors that produces AFP, such as some gastric cancer, intestinal cancer and fetal adenocarcinoma of the lung [27]. The name "gastric cancer with primitive enterocyte phenotype" is a possibly more scientific name.

Consistent with the research by Yamazawa et al., the GAPEP tumors in this study were mostly adenocarcinomas of common morphology, and these tumors had similar biological behaviors [28–30]. This suggests that the traditional pathological diagnosis of special morphology is insufficient. GPC3 is a glypican-related proteoglycan that is sensitive for diagnosing HCC, distinguishing it from benign hepatocellular lesions [10]. SALL4 is a novel sensitive and specific marker for both primary and metastatic germ cell tumors [15]. Both GPC3 and SALL4 have significance in gastric carcinoma [14]. This study further confirms that the determination of GAPEP based on the immunohistochemistry of AFP, GPC3, and SALL4 is indeed of significance for judging prognosis. Molecular research on GAPEP and its related tumors has yielded some results [31, 32]. Noboru et al. found frequent loss of SMAD4 heterozygosity by immunohistochemistry in gastric adenocarcinoma with enteroblastic differentiation [33, 34]. Yoichi et al. found that GAED frequently harbors TP53 mutations and ERBB2 amplification [35]. Fujimoto et al. found that HER2 is frequently overexpressed in hepatoid adenocarcinoma and gastric carcinoma with enteroblastic differentiation [36]. According to our previous research, GAPEP accounted for 7.53% of surgical specimens [37]. Defining this particular type may have some significance in selecting patients for more aggressive treatment [38]. However, several large-scale gastric cancer molecular typing studies, such as The Cancer Genome Atlas (TCGA) and the Asian Cancer Research Group (ACRG) [39–42], have not included GAPEP. More studies are needed on the molecular pathological features of GAPEP.

There were still some weaknesses in this research. GA with AFP-producing and GAPEP were rare tumor, and the quantity of those cases was limited. More evidences are in need to confirm the results in future. And we couldn’t establish the comparator arm in group B because the complex reasons for not getting operation. The two methods both have their own advantages, the serological method is noninvasive and specific. However, the results may be affected by other factors (liver disease, germ cell tumors, etc.). Although immunohistochemical examination is invasive, it is sensible and can better reflect the nature of the tumor. In applying both methods to determine the prognosis of a patient, the AUC was slightly better than using only one of the two. Therefore, we recommend using sAFP and AFP/GPC3/SALL4 immunohistochemistry for all cases of gastric cancer.

## Conclusions

Both the serum AFP level and immunohistochemical expression of can be used to indicate a poor prognosis for gastric adenocarcinoma. The serum AFP level (especially > 1000 ng/mL) is more specific, and immunohistochemistry is more sensitive.

## Data Availability

The datasets used and/or analysed during the current study available from the corresponding author on reasonable request.
